# Effective delivery of osteopontin small interference RNA using exosomes suppresses liver fibrosis *via* TGF-β1 signaling

**DOI:** 10.3389/fphar.2022.882243

**Published:** 2022-09-02

**Authors:** Min Tang, Cheng Guo, Mengxue Sun, Hao Zhou, Xin Peng, Jianli Dai, Qin Ding, Ying Wang, Changqing Yang

**Affiliations:** ^1^ Department of Gastroenterology and Hepatology, Digestive Disease Institute, Tongji Hospital, Tongji University School of Medicine, Shanghai, China; ^2^ Endoscopy Center, Department of Gastroenterology, Shanghai East Hospital, School of Medicine, Tongji University Shanghai, China; ^3^ Affiliated Hangzhou Chest Hospital, Zhejiang University School of Medicine, Hangzhou, China; ^4^ Tianjin Key Laboratory of Technologies Enabling Development of Clinical Therapeutics and Diagnostics, School of Pharmacy, Tianjin Medical University, Tianjin, China; ^5^ Biology Department of Pharmaron Beijing Co., Ltd., Beijing, China; ^6^ Nutrition Department, Shanghai Pulmonary Hospital Affiliated to Tongji University, Shanghai, China; ^7^ Department of Infection Management, The Affiliated Suzhou Hospital of Nanjing Medical University, Suzhou Municipal Hospital, Gusu School, Nanjing Medical University, Suzhou, China

**Keywords:** liver fibrosis, osteopontin, small interfering RNA, exosome-mediated delivery, TGF-β1 signaling

## Abstract

**Objective and aims:** Osteopontin (OPN), an oxidant stress sensitive cytokine, plays a central role in liver fibrosis. While OPN expression can be reduced by small interfering RNA (siRNA), the challenge to deliver siRNA safely and effectively into liver remains unresolved. Exosomes are promising natural nanocarriers for drug delivery that are able to enter cells with different biological barriers efficiently. In this study, we used exosomes as a delivery vehicle to target OPN in liver fibrosis.

**Methods:** Exosomes selectively home to fibrotic liver according to small animal imaging system. Electroporation technique was used to engineer exosomes to carry siRNA targeting OPN (Exo^siRNA−OPN^). Primary hepatic stellate cells (HSCs) were isolated and treated with Exo^siRNA−OPN^ to assess the effect on activated HSCs (aHSCs). Immunofluorescence for α−SMA, an aHSCs marker, and sirius red staining were performed to assess ECM deposition. Finally, plasma OPN from patients with liver fibrosis was identified by ELISA assay.

**Results:** Exosome-mediated siRNA delivery systems show high uptake and low toxicity. Besides, Exo^siRNA−OPN^ suppressed HSCs activation and ECM deposition and more efficiently improved liver function when compared to naked siRNA-OPN. Moreover, Exo^siRNA−OPN^ was assumed inhibiting TGF-β1 signaling activation, along with other fibrotic-related genes based on a GEO datasheet of liver fibrosis samples for correlation analyzes. Exo^siRNA−OPN^ inhibited TGF-β1 signaling by decreasing high-mobility group box-1 (HMGB1). Plasma proteins from chronic HBV-induced fibrosis patients were identified that patients with high OPN expression correlates with more advanced fibrosis progression.

**Discussion:** This study shows that exosome-mediated siRNA-OPN delivery may be an effective option for the treatment of liver fibrosis.

## Introduction

Liver fibrosis presents an extensive unmet clinical challenge. Currently, there is no proven anti-fibrotic treatment that reverses or halts the progression of liver fibrosis. Liver fibrosis is a process of excessive extracellular matrix (ECM) accumulation in the liver caused by chronic liver injury of a variety of etiologies, including autoimmune hepatitis, hepatitis B and C, alcoholic steatohepatitis, and non-alcoholic liver disease (Schwabe and Bataller, 2015; Wu et al., 2019; Ginès et al., 2021a). Liver fibrosis has the potential to progress to cirrhosis, which is the end stage of chronic liver disease. If not prevented, patients with liver fibrosis may be at risk of progressing to hepatocellular carcinoma and liver failure (Ginès et al., 2021b), for which the only therapy remaining is liver transplant. Therefore, a better understanding of the molecular mechanism involved in liver fibrosis would facilitate to develop preventive approaches for liver fibrosis, and effective and feasible treatments for liver fibrosis urgently need innovative approaches.

Activated hepatic stellate cells (HSCs) are principal cell source responsible for ECM accumulation, which is the central event underlying liver fibrosis (Chen et al., 2015; Tsuchida and Friedman, 2017). During liver injury, a variety of growth factors and fibrogenic cytokines are stimulated, the quiescent HSCs transdifferentiate and proliferate into myofibroblast-like cells that secret ECM-related proteins and tissue inhibitors of matrix metalloproteinases (TIMPs), resulting in liver fibrosis (Friedman, 2008; Kisseleva et al., 2012). Therefore, the key step to inhibit liver fibrosis is to promote the apoptosis of HSCs or inhibit the activation of HSCs (Lok and Lyle, 2019). Transforming growth factor−β1 (TGF−β1) is believed to be the most potent fibrogenic cytokine, stimulating HSCs activation (Tsukamoto et al., 2011; Chen et al., 2021). It is reported that strategies targeting at disrupting TGF−β1 synthesis and/or signaling pathways markedly reduce liver fibrosis (Li et al., 2021; Sun et al., 2021; Yang et al., 2021).

Osteopontin (OPN), a matrix-bound protein, plays an important role in liver fibrosis (Song et al., 2021). Following liver injury, OPN was secrered, leading to an increase of serum OPN (Lorena et al., 2006; Tang et al., 2020). Collagen formation is markedly decreased in fibrotic liver after antagonizing OPN (Matsue et al., 2015). In addition, plasma OPN is considered to be a potential prognostic indicator for the advanced liver fibrosis (Matsue et al., 2015). Serum OPN levels progressively increase from stage 0–4 in fibrotic patients with hepatitis B and C (Sobhy et al., 2019). A clinical trial shows plasma OPN is enhanced in NAFLD-induced fibrosis (NCT00794716) (Glass et al., 2018). Also, serum OPN is proved a promising biomarker for the severity of NASH-induced and alcoholic-associated cirrhosis (Syn et al., 2012; Simão et al., 2015). OPN was reported to activate HSCs and induce ECM deposition through a TGF-β1-dependent signaling pathway (Xiao et al., 2012). OPN can promote the activation of HSCs by binding to its receptor integrin αvβ3, and antagonizing integrin αvβ3 can reverse the effects caused by OPN. Although OPN has emerged as a potential target for the treatment of liver fibrosis, it still remains an unresolved challenge due to the lack of specific OPN inhibitors.

RNA interference (RNAi) therapy in gene regulation is emerging as one of the promising drugs for the treatments of a wide array of diseases. However, its clinical application is limited owing to their inefficient and ineffective delivery to target tissue and cells (Zhou et al., 2016). Herein, innovatively designed nanocarriers for the delivery of oligonucleotides for successful RNAi therapeutics are needed. In this regard, exosomes, secreted by most cells, are nanosized extracellular vesicles ranging in size typically from 40 to 150 nm. The most common exosomal proteins are fusion proteins and membrane transporters (flotillin, Annexins and GTPases), heat shock proteins, tetraspanins (CD9, CD63 and CD81), MVB synthesis proteins (TSG101 and Alix) (Kamerkar et al., 2017; Kalluri and LeBleu, 2020; Tang et al., 2021a). Exosomes commonly exist in biological fluids such as plasma, urine, cerebrospinal fluid, amniotic fluid and have the intrinsic ability to traverse biological barriers, which highlight their role in cell-to-cell communication and transferring bioactive molecules such as proteins and RNA (Kamerkar et al., 2017; Kalluri and LeBleu, 2020; Tang et al., 2021a). Thus, Exosomes represent a novel and exciting delivery tool for the field of RNAi therapeutics.

Mesenchymal stem cells (MSCs), one of the most easily accessible primary cells, possess several advantages as an exosome source over other cell types (Yu et al., 2014; Cone et al., 2021; Psaraki et al., 2021). Recently, MSCs derived exosomes were reported as carriers for the delivery of siRNA (Tang et al., 2021a; Huang et al., 2021). MSC-derived exosomes likely have multiple features that enable them as natural nanocarriers for therapy and are under active research (Luan et al., 2017). In our study, we tested whether an antifibrotic strategy based on exosomes in liver fibrosis could be employed to target OPN specifically and the underlying mechanism. We engineered exosomes containing siRNA-OPN and evaluated their therapeutic role in carbon tetrachloride (CCl_4_)-induced murine model of liver fibrosis (Kisseleva and Brenner, 2021). We demonstrate that administration of iExosomes containing siOPN attenuate progression of liver fibrosis effectively and provide a therapeutic option of exosome-based therapy in liver fibrosis.

## Methods

### Gene expression profiling data

The gene expression datasets analyzed were retrieved from the National Center for Biotechnology Information Gene Expression Omnibus database in this study (https://www.ncbi.nlm.nih.gov/geo/). After careful review, two gene expression profiles (GSE55747, https://www.ncbi.nlm.nih.gov/geo/query/acc.cgi?acc=GSE55747 and GSE71379, https://www.ncbi.nlm.nih.gov/geo/query/acc.cgi?acc=GSE71379) were chosen for analysis in CCl_4_-induced liver fibrosis. Data from these two datasets were further standardized and analyzed with Rstudio (v.1.4.1106).

### Data processing of differentially expressed genes

GEO2R (https://www.ncbi.nlm.nih.gov/geo/geo2r) was analyzed to detect the DEGs between fibrotic liver and healthy liver samples. Genes which met the cutoff criteria with adjusted *p* < 0.05 were considered as DEGs. The heatmap and volcano were painted to show the DEGs by using the “ggplot2” R package (Ito and Murphy, 2013).

### Construction of a protein-protein interaction network

To assess the interactions between OPN-ECM, the Search Tool for the Retrieval of Interacting Genes (STRING) database (https://string-db.org/) was used to explore the PPI interaction networks. A PPI network was generated using interactors with a combined confidence score of ≥0.4, and the combined interaction pairs of these DEGs were downloaded from STRING. To further assess the target gene of OPN, we assessed the correlation between OPN and selected ECM-related genes based on the connection degree. Finally, TGF-β1 was found to be the top one.

### Mice

The 6-week-old male C57BL/6 mice, obtained from Shanghai JieSiJie Laboratory Animal Co.,Ltd., were randomly divided into two groups, including the control group and liver fibrosis group. The mice model of liver fibrosis was induced by intraperitoneal injection of 10% of CCl_4_ (Sigma, United States) diluted in olive oil twice a week for 4 weeks. In control group, olive oil was injected intra-peritoneally twice a week for 4 weeks.

For the exosomes treatment experiments, the sham group was received 100 μL olive oil for 37 days. Other mice were administered with 10% CCl_4_ and randomly assigned for 5 groups after CCl_4_ treatment for 7 days. Mice were also injected with 5 μg siRNA (RiboBio, China) of 1*10^9^ engineered exosomes i.v., or 5 μg siRNA alone in PBS (100 μL) every other day for 30 days. After the last treatment, the mice were euthanized in 24 h. The protocol was approved by and in accordance with the guidelines of the Animal Experiment Committee of Tongji Hospital.

### Exosomes biodistribution in mice

For exosomes biodistribution, 1 μM DiR (Perkin Elmer) was added per 12 billion exosomes, and incubated at for 1 h at the temperature of 37 and 4°C for 15 min, and then ultracentrifuged for 3 h at 100,000 x g. 5*10^9^ labeled exosomes were washed for 3 times and then resuspended in PBS (100 μL). Six hours after injection, organs listed were collected and imaged immediately with the *in Vivo* Imaging Systems (IVIS) 200 small animal imaging system.

### Exosome uptake experiment

Exosomes were labeled with Dil (1 μM, Beyotime). The DiI-exosome suspension was incubated at 37°C for 1 h. Excess dye was removed by ultracentrifugation (Beckman Coulter) at 100,000 g for 1 h at 4°C, and the pellets were washed in PBS for 3 times. PBS was used to dilute the final pellets. The labeled-exosomes were added in the supernatant HSCs for 24 h. The uptake was observed by confocal microscopy.

### Immunofluorescence analyses

Frozen sections were blocked for one hour with 1% BSA and then incubated overnight with anti *α*-SMA (proteintech, 1:200), OPN (Servicebio, 1:50), HMGB1 (Servicebio, 1:100) and TGF-β1 (Servicebio, 1:100).

Immunofluorescence was qualified as percentage of positive cells using the counting tool of Adobe Photoshop 7.0.

### Statistical analyses

All details associated with statistical analyses are in the figure legends. Values are shown in this study as mean ± SD. *p* < 0.05 was considered statistically significant. Statistical significance was established using GraphPad Prism software. Among groups, Welch ANOVA with Dunnett’s T3 post–hoc and Brown-Forsythe analysis was used for values with significantly different SDs or one-way ANOVA with Sidak’s post–hoc analysis was used for values with similar SDs. When differences between the two groups were compared, unpaired two–tailed Student’s t–test or Welch’s unpaired two-tailed *t*-test for data with significantly different SDs were used. *P* < 0.0001 ****, *p* < 0.001 ***, *p* < 0.01 **, *p* < 0.05 *.

## Results

### Accumulation of OPN during progression of liver fibrosis

**FIGURE 1:**
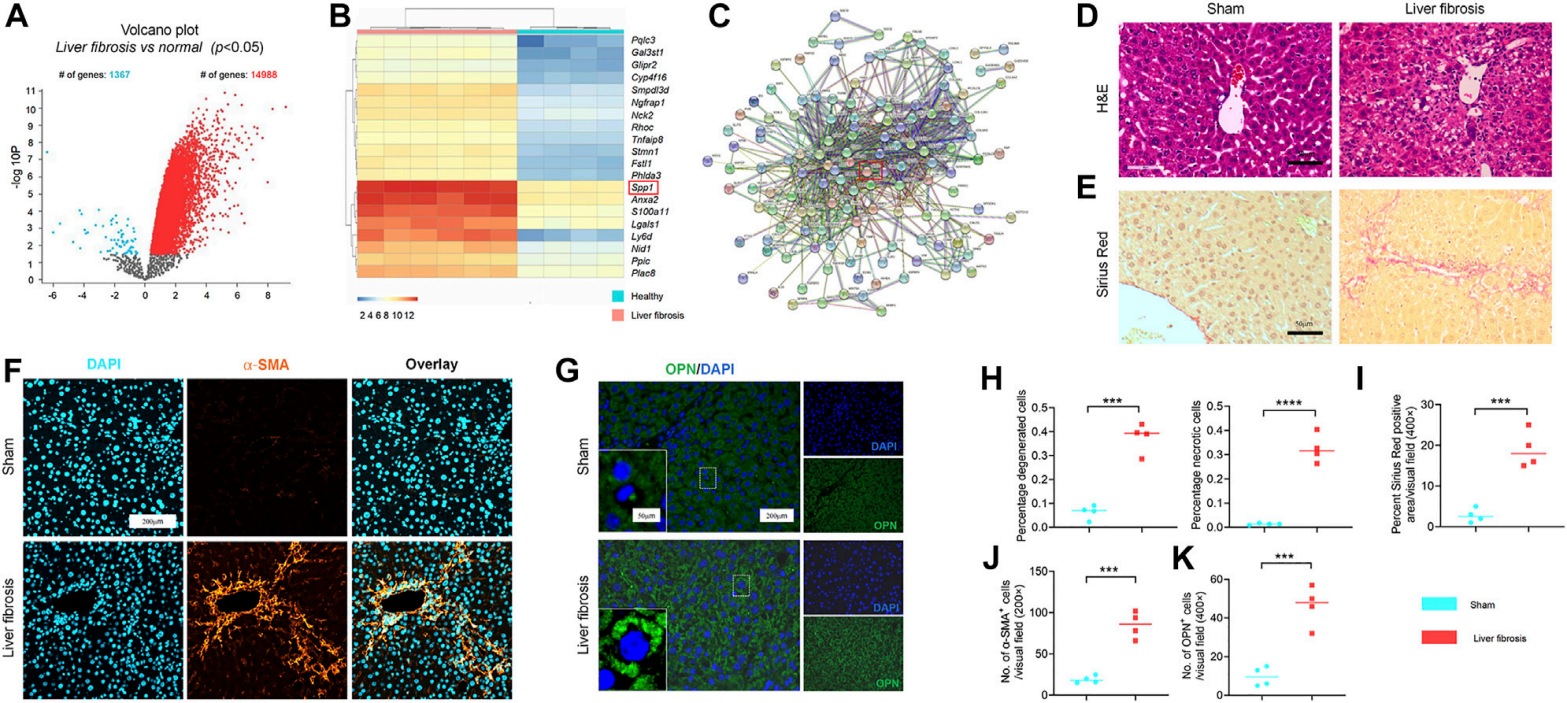
Accumulation of OPN during progression of liver fibrosis murine model. **(A)** Volcano plots showing the number of differentially expressed genes (DEGs) in GSE55747; **(B)** Heatmap for the top 20 DEGs in GSE55747. **(C)** Protein-protein interaction networks (PPI) of OPN and ECM-related genes according to GSE71379. **(D,H)** Representative H&E staining **(D)** of liver sections from CCl_4_-treated and olive oil vehicle-treated mice. **(H)** Percentage of degenerated and necrotic hepatocytes per visual field. *n* = 4 mice per group; Scale bar: 50 μm. **(E,I)** Sirius red staining **(E)** in each group with indicated treatments. **(I)** Percent sirius red positive areas per visual field (%). *n* = 4 mice per group; Scale bar: 50 μm. **(F,G,J,K)** α−SMA **(F)** and OPN **(G)** immunofluorescence (IF) staining in mice (5 visual fields for each tissue analyzed). The average number of α−SMA **(J)** and OPN **(K)** positive cells. *n* = 4 mice per group; Scale bar: 200 μm. Data in Figure 1 are expressed as mean ± SD. Individual dots represent distinct mice in graphs. Unpaired t–test was used in fig **(D–G,J,K)**. *p* < 0.001 ***, *p* < 0.0001 ****.

**FIGURE 2:**
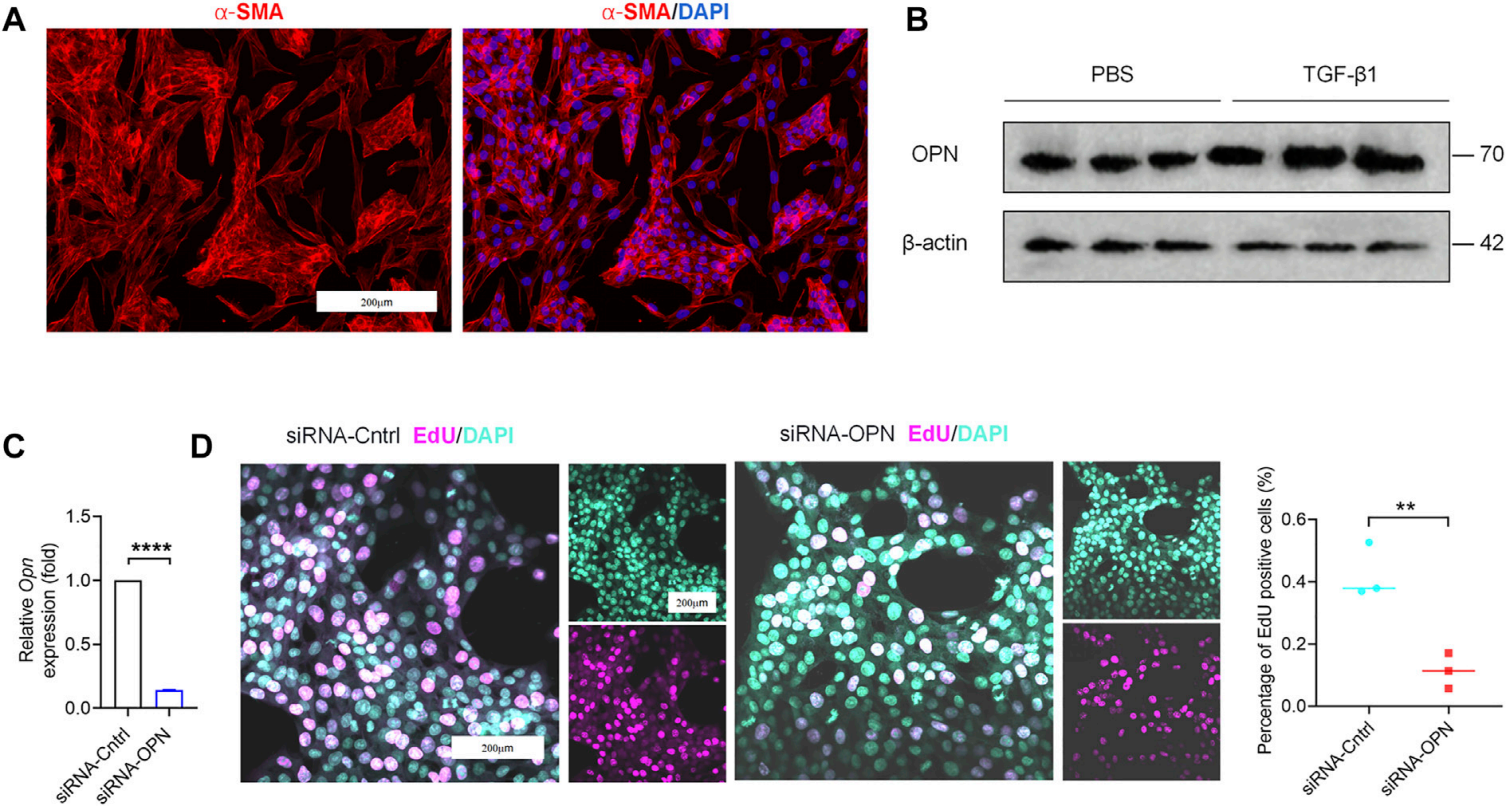
Accumulation of OPN activated primary HSCs. **(A)** Primary HSCs were isolated from wild-type (WT) mice. Images of IF staining for α−SMA (red) and DAPI (blue) of HSCs. Scale bar: 200 μm. **(B)** OPN expression at protein level by western blot analysis. **(C)** qPCR analysis of OPN in primary HSCs after transfection with siRNA. **(D)** Proliferated cells detected by staining of EdU (left panel). The percentages of EdU-positive cells were quantified (right panel). Scale bar: 200 μm. Data are expressed as the mean ± SD. **(C,D)** unpaired t–test. *P* < 0.01 **, *p* < 0.0001 ****.

Microarray data, obtained from GSE55747 and GSE71379, were used to analyze mRNA levels in CCl_4_ model of liver fibrosis and healthy mice. Based on the criteria of adjusted *p* < 0.05, 16355 DEGs totally were identified from GSE55747, including 1367 downregulated genes and 14988 upregulated genes according to volcano plot (Figure 1A). Of the top 20 DEGs, OPN was further found to be the most up-regulated gene (Figure 1B). Besides, OPN was associated with canonical fibrosis-related genes in GSE71379, including Col1a1 and Col1a2 (Figure 1C). Therefore, we hypothesized that OPN played an important role in the progression of liver fibrosis and maybe one of the most potentially target in the treatment in liver fibrosis. To test our hypothesis, we constructed CCl_4_-induced liver fibrosis model (Figures 1D–F,H–J) to evaluate the role of OPN in liver fibrosis. To this end, we conducted comparison of the expression abundance of OPN expression in fibrotic liver, as well as in activated primary HSCs. Immunofluorescence (IF) staining of OPN in fibrotic liver confirmed that OPN (Figures 1G,K) was significantly more highly expressed in fibrotic liver along with degenerated hepatocytes accumulation (Figures 1D,H), collagen deposition (Figures 1E,I) and HSCs activation (Figures 1F,J). Next, primary HSCs were isolated from wild-type (WT) mouse and activated spontaneously in culture on plastic for 7 days (Lu et al., 2019; Tang et al., 2021a) or activated with TGF−β1 stimulation. aHSCs were characterized by gain of α−SMA expression (Figure 2A). OPN was accumulated in aHSCs, which is consistent with the data analysis from GSE55747 (Figure 2B). Then we knocked down OPN in primary HSCs (Figure 2C), and measured the proliferation of HSCs to assess the activation by EdU analysis (Figure 2D). As a result, after OPN was down-regulated, activation of HSCs was inhibited. These supporting that OPN is an important mediator in liver fibrosis.

### Isolation and identification of exosomes released and up-taken by primary HSCs

**FIGURE 3:**
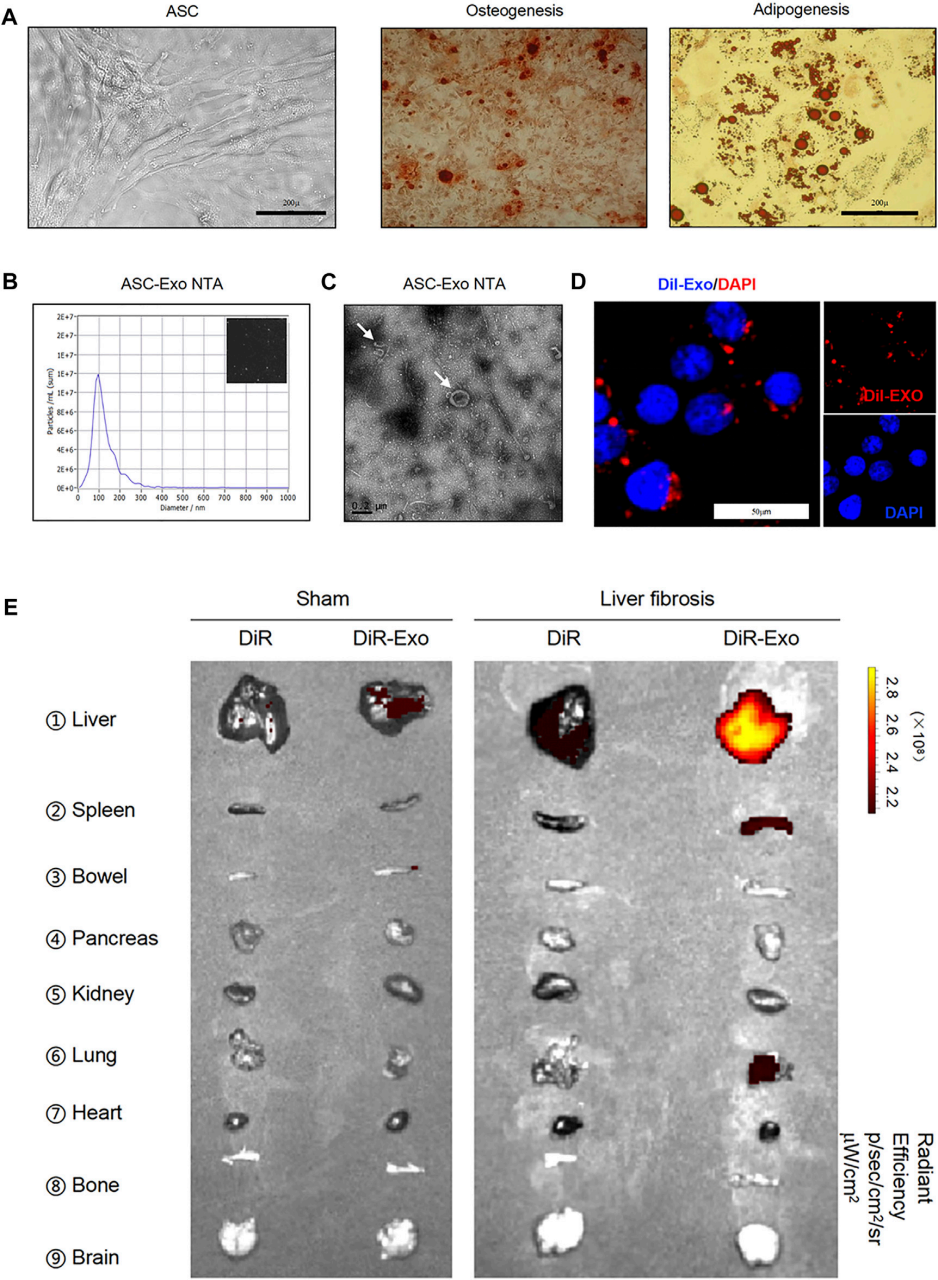
Isolation and identification of exosomes released and up-taken by primary HSCs. **(A)** Images of human adipose tissue-derived mesenchymal stem cells (ASCs) (left panel). Two groups of ASCs were subjected to osteogenic (middle panel) or adipogenic differentiation medium (right panel) and stained with Alizarin Red S or Oil Red O, respectively. Scale bar: 200 μm. **(B)** Nanosight imaging of isolated exosomes. **(C)** Appearance of exosomes by transmission electron microscopy. Scale bar: 0.2 μm. **(D)** Primary HSCs were incubated for 24 h with Dil labeled-exosomes. Cells were visualized for exosome fluorescence (red) and DAPI (blue) by confocal microscopy. **(E)** Images of isolated organs analyzed for presence of DiR-exosomes in sham (left panel) and liver fibrosis (right panel).

Adipose tissue-derived MSCs (ASCs) represent a highly advantageous tool for stem cell-based therapy (Lou et al., 2015; Tang et al., 2019; Lou et al., 2020). Under specific-differentiation conditions *in vitro*, ASCs can differentiate into adipocytes and osteoblasts depending on the presence of adipogenic and osteogenic factors (Tang et al., 2019). Positive staining of Alizarin Red S or Oil Red O was observed after osteogenic induction for 21 days or adipogenic induction for 7 days, respectively, as we previously reported (Figure 3A). To determine the characteristic ASC-derived exosomes (ASC-Exo), exosomes were extracted from the supernatant of ASCs. Nanoparticle tracking analysis (NTA) showed size distribution with a mean diameter, which were mostly ranged between 40 and 150 nm (Figure 3B), and transmission electron microscope (TEM) showed the classic exosomal morphology (Figure 3C). Besides, CD81 and CD63, the markers of exosomes, were detected by western blot (Supplementary Figure S1). Next, DiI was utilized to label exosomes. We observed with laser scanning confocal microscope that red coated exosome can be taken up by HSCs efficiently. These indicate that we have successfully isolated exosomes from ASCs, and demonstrated that they can be up-taken by HSCs (Figure 3D).

Further, we explored the *in vivo* biodistribution of exosomes, DiR-exosomes or DiR as control were administered *i.v.* into WT and fibrotic mice induced by CCl_4_, a widely used experimental model. The results showed that exosome accumulated in the normal liver and spleen, and lower signal detected in the bowel, kidney and pancreas (Figure 3E). However, higher enrichment of exosome-associated signal was detected in the fibrotic liver when compared with the normal liver (Figure 3E), suggesting that exosome can reach to the liver efficiently, and probably provide lower systemic toxicities.

### iExosomes targeting OPN inhibit liver fibrosis

**FIGURE 4:**
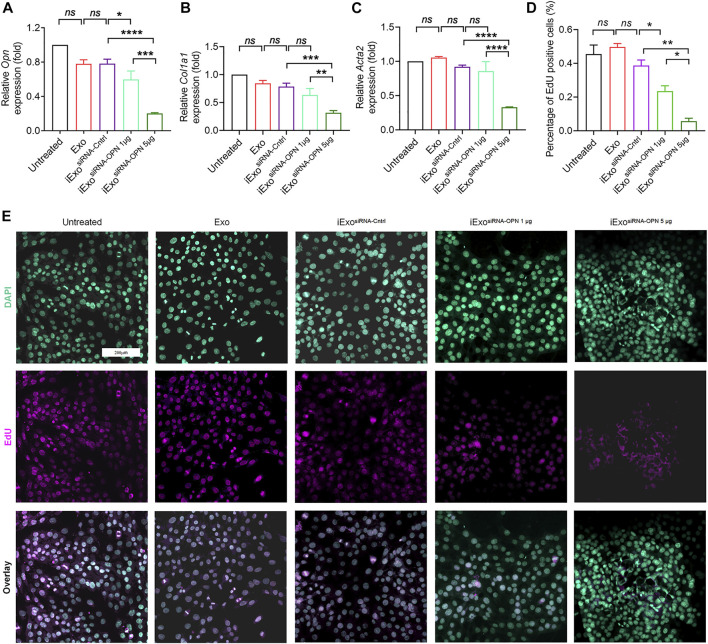
iExosomes targeting OPN inhibit liver fibrosis *in vitro*. **(A)** *Opn* expression by qPCR analysis in primary HSCs treated with iExosomes electroporated with 1 μg or 5 μg siRNA-OPN. n = 3 independent experiments. **(B)** *Col1a1* expression by qPCR analysis in primary HSCs subjected to listed treatment. *n* = 3 independent experiments. **(C)** *Acta2* expression in primary HSCs with the indicated treatment by qPCR analysis. *n* = 3 independent experiments. **(D,E)** Proliferate cells stained with EdU **(E)** and the percentage of positive cells **(D)** to total cells in primary HSCs in each group. *n* = 3. The data in figure **(A–C)** are presented as One-way ANOVA with Sidak’s post–hoc analysis; Brown-Forsythe and Welch ANOVA with Dunnett’s T3 post-hoc analysis were used in fig **(D)**. *p* < 0.05 *, *p* < 0.01**, *p* < 0.001 ***, *p* < 0.0001 ****.

**FIGURE 5:**
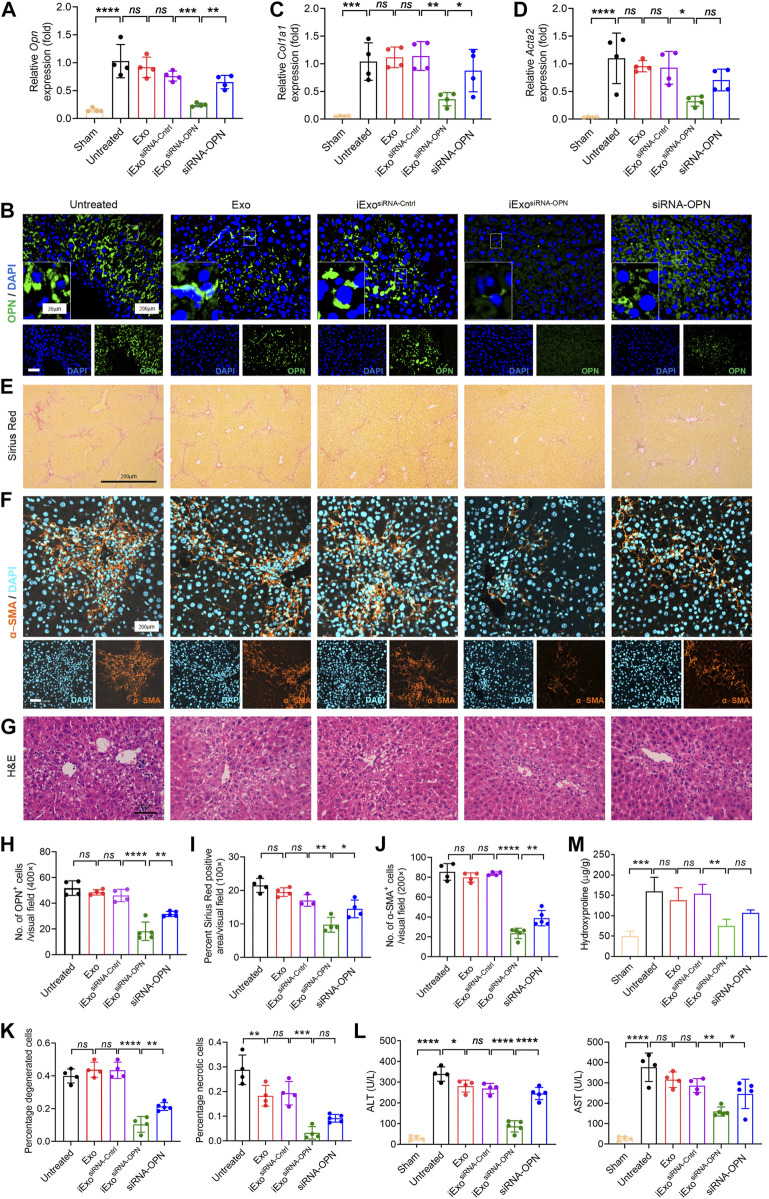
iExosomes targeting OPN inhibit liver fibrosis *in vivo*. **(A,B,H)** Relative *Opn* expression analyed by qPCR analysis **(A)** and IF staining **(B)**. **(H)** The average number of OPN positive cells in each visual field. Untreated, Exo, and iExo^siRNA−Cntrl^: *n* = 4 mice per group; iExo^siRNA−OPN^, siRNA-OPN: *n* = 5 mice per group. Scale bar: 200 μm (inserted scale bar: 50 μm). **(C,D)** *Col1a1* **(C)** and *Acta2* **(D)** expression by qPCR analysis in liver from mice subjected to exosomes electroporated with siRNA or nake siRNA. *n* = 4 mice per group. **(E,I)** Sirius red staining **(E)** of listed liver sections. **(I)** Percentage of sirius red positive area in each visual field. Untreated, Exo, and iExo^siRNA−Cntrl^: *n* = 4 mice per group; iExo^siRNA−OPN^, siRNA-OPN: *n* = 5. Scale bar: 200 μm. **(F,J)** α−SMA IF staining **(F)** in each group. **(J)** The average number of α−SMA positive cells in each visual field. Untreated, Exo, and iExo^siRNA−Cntrl^: *n* = 4 mice per group; iExo^siRNA−OPN^, siRNA-OPN: *n* = 5. Scale bar: 200 μm. **(G,K)** H&E staining **(G)** of liver with the indicated treatment. **(K)** Percentage of necrotic and degenerated hepatocytes. Untreated, Exo, and iExo^siRNA−Cntrl^: *n* = 4 mice per group; iExo^siRNA−OPN^, siRNA-OPN: *n* = 5. Scale bar: 200 μm. **(L)** Serum ALT (left panel) and AST (right panel) levels in the listed groups. Untreated, Exo, and iExo^siRNA−Cntrl^: *n* = 4 mice per group; iExo^siRNA−OPN^, siRNA-OPN: *n* = 5 mice per group. **(M)** Hydroxyproline content in rat livers. *n* = 3 mice per group. The data are expressed as Sidak’s post–hoc analysis. *P* < 0.05 *, *p* < 0.01 **, *p* < 0.001 ***, *p* < 0.0001 ****.

Since OPN knock-down could inhibit fibrogenesis, which is important in the treatment in liver fibrosis, therefore, we next explored the therapeutic efficacy of iExosomes targeting OPN as anti-fibrotic agent. Exosomes were electroporated with two different dosage of siRNA-OPN, 1 billion exosomes electroporated with 1 μg (iExo^siRNA−OPN 1μg^) or 5 μg siRNA (iExo^siRNA−OPN 5μg^). iExosomes with siRNA-OPN were used to treat HSCs, controlled by untreated HSCs and HSCs treated with exosomes, or exosomes containing control siRNA. After pre-treatment, HSCs were harvested for fibrogenesis and proliferation assay. As the result shown, compared with the control group, the expression of OPN (Figure 4A), fibrosis-associated genes (Figures 4B,C) and proliferation (Figures 4D,E) of HSCs treated with iExosomes containing with siRNA-OPN were significantly down-regulated. What’s more, superior efficacy was observed at iExo^siRNA−OPN 5μg^ compared to iExo^siRNA−OPN 1μg^. In other word, iExosomes could inhibit HSCs activation efficiently, especially at the dosage of 5 μg siRNA-OPN. Based on these, we conducted *in vivo* experiment with *i.v.* injection CCl_4_-induced liver fibrosis models of iExo^siRNA−OPN 5μg^. Additionally, to investigate whether iExosomes had enhanced effect on liver fibrosis, when compared with naked siRNA-OPN, we also treated mice with siRNA-OPN alone at the same dosage of iExo. Chronic exposure to the hepatotoxin CCl_4_ induces liver damage and necrosis, which drive accumulation of aHSCs and progressive fibrosis (Mederacke et al., 2013). HE staining was used to analyse the necrotic and degenerated hepatocytes, while sirius red staining was used to assess collagen deposition. Results showed that a significant reduction in OPN expression (Figures 5A,B,H), as well as *Col1a1* (Figure 5C) and *α-Sma* (Figure 5D) expression in iExo^siRNA−OPN^ group. This suggested that a significant reduction in collagen deposition and HSCs activation in mice treated with iExo^siRNA−OPN^, whereas a modest reduction in fibrogenesis was found in mice treated with siRNA-OPN alone, which was further evidenced by sirius red staining (Figures 5E,I) and IF staining of α−SMA (Figures 5F,J). Liver function, ascertained with the percentage of necrosis and degeneration in hepatocytes was markedly reduced when mice were administered with iExo^siRNA−OPN^ according to HE staining (Figures 5G,K). Similarly, the serum levels of ALT and AST, was significantly restored in mice treated with iExo^siRNA−OPN^, as well as with naked siRNA-OPN, albeit to a lesser extent (Figure 5L). As for the hepatic hydroxyproline (Hyp), we also found the similar results (Figure 5M). Besides, exosomes treatment did not cause observable cytotoxicity to other organs according to the HE staining (Supplementary Figure S2).

### iExosomes targeting OPN induced TGF-β1 signaling via HMGB1 activation

**FIGURE 6:**
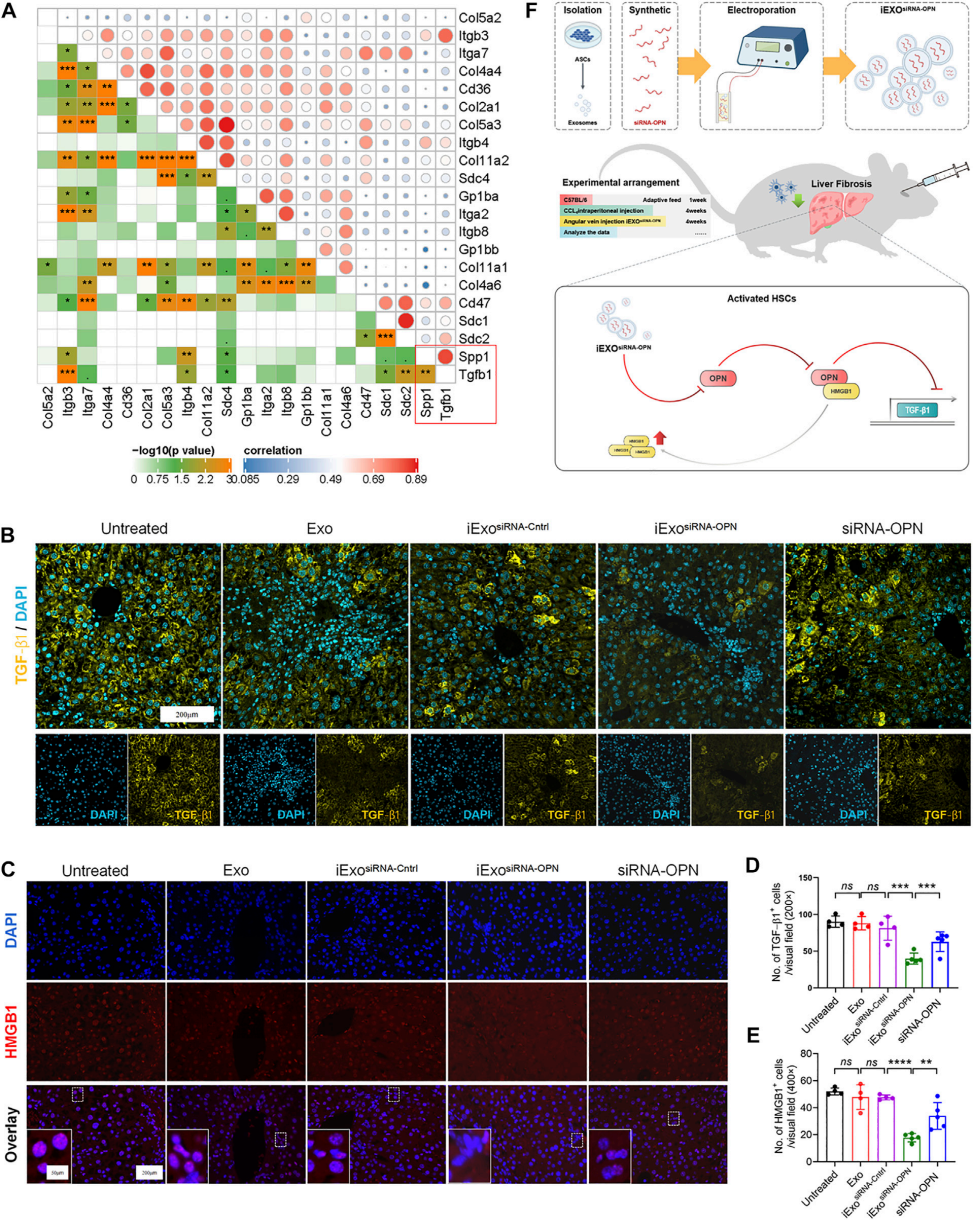
iExosomes targeting OPN induced TGF-β1 signaling *via* HMGB1 activation. **(A)** Correlation between OPN and selected ECM-related genes. **(B,D)** TGF−β1 IF staining of the listed groups and number of TGF−β1 positive cells in each visual field. Untreated, Exo, and iExo^siRNA−Cntrl^: *n* = 4 mice per group; iExo^siRNA−OPN^, siRNA-OPN: *n* = 5. **(C,E)** HMGB1 IF staining of liver section in each group and average number of HMGB1 positive cells in each visual field. Untreated, Exo, and iExo^siRNA−Cntrl^: *n* = 4 mice per group; iExo^siRNA−OPN^, siRNA-OPN: *n* = 5. **(F)** Potential working model of iExo^siRNA−OPN^ inhibiting HSCs activation and attenuating liver fibrosis progression. The data are expressed as Sidak’s post–hoc analysis. *P* < 0.001 ***, *p* < 0.01 **.

Next, we attempted to elucidate the mechanism of iExosomes on ECM deposition and fibrogenesis. First, correlation analyzes of ECM-related genes was conducted. Among those ECM-associated genes, OPN was strongly correlated with TGF-β1, a very robust driver of HSCs activation and extracellular matrix production (Figure 6A). We further determined the TGF-β1 expression with OPN knockdown by IF staining, which suggested iExosomes containing siRNA-OPN inhibiting liver fibrosis *via* regulating TGF-β1 (Figures 6B,D). However, the mechanism of how iExo^siRNA−OPN^ regulates TGF-β1 in liver fibrosis is unclear.

Interestingly, it was reported that OPN could increase HMGB1 in the cytoplasm *via* translocation from the nucleus predominantly (Arriazu et al., 2017; Tang et al., 2021b), and HMGB1 activation was correlated with TGF-β1 expression (Zou et al., 2021). Besides, HMGB1 was also inhibited in iExo^siRNA−OPN^ group as IF staining showed (Figures 6C,E). Therefore, we assumed that iExosomes containing siRNAOPN inhibited TGF-β1 expression *via* inhibiting HMGB1 translocation to cytoplasm and further inactivating HSCs and reducing liver fibrosis (Figure 6F).

### Serum OPN upregulated in human liver fibrosis

**FIGURE 7:**
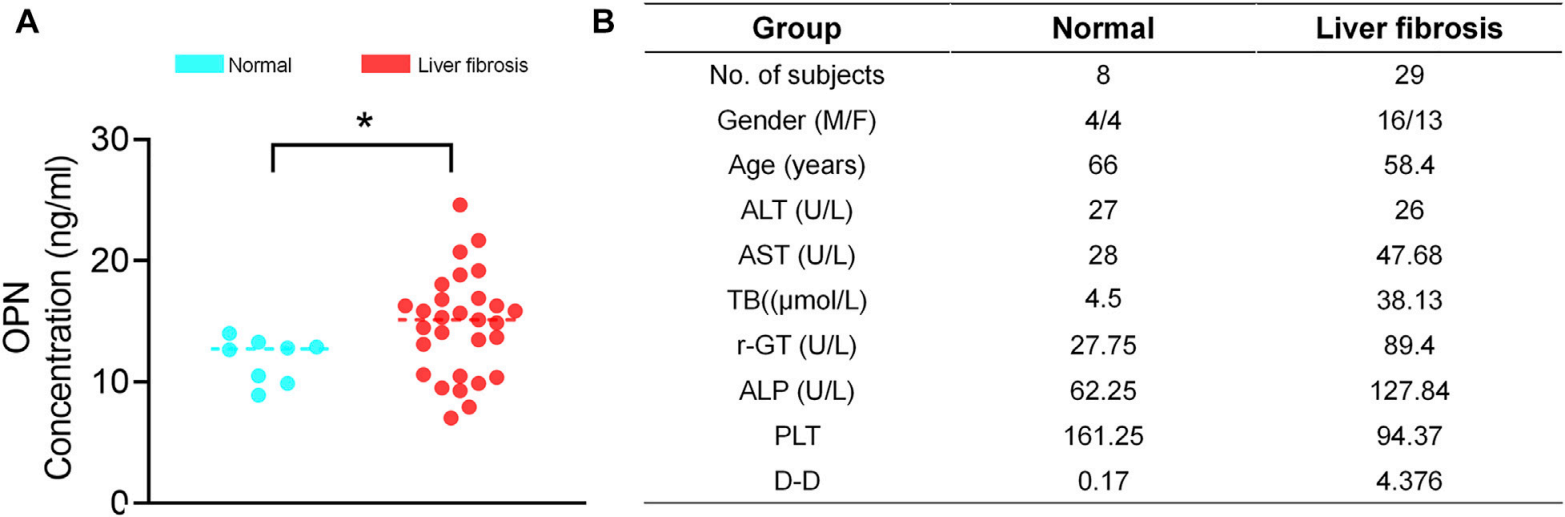
Serum OPN upregulated in liver fibrosis patients. **(A)** The serum was collected from patients with liver fibrosis and healthy people and centrifuged. The OPN concentration was measured by ELISA. **(B)** Clinical profiles of healthy people and patients with liver fibrosis enrolled for serum OPN analysis. The data are expressed as unpaired two–tailed Student’s t–test. *P* < 0.05 *.

Finally, we examined OPN expression in patients with liver fibrosis. Serum obtained from patients with liver fibrosis showed higher OPN expression than those observed in normal human (Figures 7A,B). Besides, we observed that people with higher TB and DD secrete more OPN protein. These results further indicate that OPN was associated with the progression of liver fibrosis. Inhibiting OPN effectively may be an option for treatment of liver fibrosis.

## Discussion

Here, we report on siRNA-based strategy aimed at inhibiting OPN transcriptionally expression using exosomes as a nonviral delivery platform for antifibrosis therapy. A number of studies have suggested that OPN plays a central role in the pathogenesis of liver fibrosis (Syn et al., 2011; Arriazu et al., 2017; Song et al., 2021), as it activates and strengthens invasive and wound-healing potential of HSCs (Urtasun et al., 2012). Primary HSCs isolated from WT mice have enhanced pro-fibrogenic potential than the cells from *Opn* ^−/−^ mice and overexpression OPN in HSCs by infection with an adenovirus induces collagen type-I production, indicating correlation between OPN and collagen I (Urtasun et al., 2012). Previously, we also verified that OPN drives fibrogenesis in NAFLD (Tang et al., 2021b). In our study, OPN was identified as a critical target in liver fibrosis according to the GEO data set (GSE55747, GSE71379), which is consistent with previous observations. Furthermore, we found that plasma OPN expression was associated with advanced liver fibrosis in patients. Herein, silencing OPN expression in the liver is one of the important strategies for the treatment of liver fibrosis. SiRNA-based therapy has shown great promise in treating diseases by silencing specific responsible gene expression. With the Food and Drug Administration (FDA) approval of four siRNA-based drugs recently, the potential of RNA-based therapeutics to become a milestone in pharmaceutical drug development has become a reality (Binzel et al., 2021). Yet its therapeutic effect still faces some challenges such as unspecificity in targeting, endosome trapping, and intracellular processing after delivery (Binzel et al., 2021; Ji et al., 2021), which resulted in diminishing clinical application. Thus, there is a desperate need for more efficient delivery vehicles that specifically target sites with better stability and affinity (Hoerter and Walter, 2007; Selvam et al., 2017).

We previously reported that exosome could enhance specificity of drug delivery (Tang et al., 2021a). Exosomes are relatively stable in the blood, enhancing the delivery of embedded siRNAs (Vader et al., 2016). Besides, exosomes are proved to increase the accumulation of drug in targeted organs such as the liver (Kamerkar et al., 2017; Tang et al., 2021a) and reduce damage to other organs, potentially further improving cargo delivery. Based on these, we labeled exosomes with DiR and administered it into mice. The data supported exosomes accumulated more in the liver in liver fibrosis. Next, we tested the antifibrosis efficacy and underlying mechanism of iExo^siRNA−OPN^. iExo^siRNA−OPN^ showed a markedly silence of OPN *in vitro*, and superior efficacy in reversing fibrogenic outcomes, restoring liver function and suppressing HSCs activation *in vivo*, when compared with naked siRNA-OPN. This is likely as a result of enhanced uptake of iExo^siRNA−OPN^ in fibrotic liver, when compared with siRNA-OPN alone.

Our data were consistent with the reported efficacy of exosomes in delivering a therapeutic cargo to targeted site (Kalluri and LeBleu, 2020; LeBleu and Kalluri, 2020; McAndrews et al., 2021a; McAndrews et al., 2021b). Our iExosomes approach provide direct and specific targeting and may be used to combine with siRNA targets, as we previous reported. Furthermore, exosomes-based therapeutic approaches for other disease are also being considered (Lou et al., 2020; McAndrews et al., 2021b). The stimulator of Interferon Genes (STING) pathway is important in both oncogenesis and cancer treatment (Motedayen Aval et al., 2020; Wu et al., 2020), and iExosomes containing STING agonists showed a markedly superior antitumor effect (Lou et al., 2020). Besides, transformed hepatocytes rely on STAT3 expression in hepatocellular carcinoma (Jung et al., 2017), and iExosomes targeting STAT3 in liver could also offer benefits in attenuating progression (Lou et al., 2020). Further, iExosome engineered to carry siRNA or short hairpin RNA (shRNA) specific to oncogenic KrasG12D has enhanced efficacy in suppressing tumorigenesis and increasing overall survival. Collectively, our research supports the therapeutic option of exosome-based therapy in liver fibrosis for clinical use.

We further selected 20 ECM-related genes based on published studies (An et al., 2017; Garcia et al., 2019; Tang et al., 2021a) and analyzed their relationship with OPN expression in fibrotic liver (GSE71379). Ultimately, we focused on TGF-β1, a typical profibrogenic cytokine, significantly decreased by iExo^siRNA−OPN^. OPN expression is required for TGF-β1-dependent myofibroblast differentiation *via* translocation of HMGB1 from the nucleus to the cytoplasm in primary cardiac fibroblasts. Coincidently, OPN was identified to be the upstream of HMGB1 involved in liver fibrosis (Arriazu et al., 2017), which is accordance with our previous finding that OPN could induce HMGB1 in HSCs activation. Combined with another literature indicating that HMGB1 might induce TGF-β1 expression in liver fibrosis (Vicentino et al., 2018), as well as our experiment data, we proposed that iExo^siRNA−OPN^ could down-regulate TGF-β1 expression *via* HMGB1. This novel finding adds more information to how OPN mediates the fibrosis progression.

## Conclusion

Overall, our work elucidated the important role of OPN in liver fibrosis and verified exosome-mediated siRNA-OPN delivery may be an effective treatment option for liver fibrosis.

## Data Availability

The datasets presented in this study can be found in online repositories. The names of the repository/repositories and accession number(s) can be found in the article/Supplementary Material.
