# Supplementary material for: Effective delivery of osteopontin small interference RNA using exosomes suppresses liver fibrosis via TGF-β1 signaling
Source: Front Pharmacol. 2022 Sep 2;13:882243. doi: 10.3389/fphar.2022.882243 (PMC9478741; doi:10.3389/fphar.2022.882243)
Supplement: Supplementary file 1 [file DataSheet1.doc]

**Effective delivery of osteopontin small interference RNA using exosomes suppresses liver fibrosis via TGF-β1 signaling**

Min Tang^1#^, Cheng Guo^2#^, Mengxue Sun^1#^, Hao Zhou^3^, Xin Peng^4^, Jianli Dai^5^, Qin Ding^6^, Ying Wang^7^*, Changqing Yang^1^*

1. Department of Gastroenterology and Hepatology, Digestive Disease Institute, Tongji Hospital, Tongji University School of Medicine, Shanghai 200065, China;
2. Endoscopy Center, Department of Gastroenterology, Shanghai East Hospital, School of Medicine, Tongji University , Shanghai, 200120, China.
3. Affiliated Hangzhou Chest Hospital, Zhejiang University School of Medicine, Hangzhou 310000, China;
4. Tianjin Key Laboratory of Technologies Enabling Development of Clinical Therapeutics and Diagnostics, School of Pharmacy, Tianjin Medical University, Tianjin, China;
5. Biology department of Pharmaron Beijing Co., Ltd. (China);
6. Nutrition Department, Shanghai Pulmonary Hospital Affiliated to Tongji University, Shanghai, China;
7. Department of Infection Management, The Affiliated Suzhou Hospital of Nanjing Medical University, Suzhou Municipal Hospital, Gusu School, Nanjing Medical University, Suzhou, China.

^#^ These authors contributed equally to this work

***To whom correspondence should be addressed:**

Changqing Yang, Department of Gastroenterology, Tongji Hospital, School of Medicine, Tongji University, Shanghai, China; Email: [cqyang@tongji.edu.cn](mailto:cqyang@tongji.edu.cn);

Ying Wang, Department of Infection Management, The Affiliated Suzhou Hospital of Nanjing Medical University, Suzhou Municipal Hospital, Gusu School, Nanjing Medical University, No. 242, Guangji Road, Suzhou 215008, China; Tel: 86-512-87236451, Email: wangy830224@sina.com

**Experimental procedures**

**1. HSCs isolation**

Male mice (C57BL/6, 6-week-old; Shanghai JieSiJie Laboratory Animal Co.,Ltd.) were used to isolate primary HSCs according to our established protocol (1). Hepatic stellate cells (HSCs) were cultured in Dulbecco’s modified Eagle medium (DMEM, Sigma) supplemented with high glucose and 20% fetal bovine serum (FBS, Biological Industries) and 1% penicillin/streptomycin (PS, Corning). HSCs were cultured for 7 days for activation and immunostained with α-SMA antibody (CST, 19245T, 1:1000) for 24 hours. HSCs images were acquired using confocal microscopy. Confocal images of immunolabeled sections were captured using either a Zeiss Axioskop 2.

**2. Western blot analysis**

The protein was extracted using the protein extraction solution and quantified by a BCA protein assay kit. Equal amounts of protein were electrophoresed on SDS-PAGE gels and transferred to PVDF membranes. The PVDF membranes were incubated overnight at 4℃ with primary antibodies, then incubated with secondary antibody for 1 hour. The primary antibodies included: CD63, CD81, OPN, β-actin. All antibodies were obtained from proteintech.

**3. RNA isolation and quantitative real-time PCR**

Total RNAs was extracted from cells or the liver tissue using the Trizol reagent (Invitrogen) and then cDNA was reversely transcribed using a reverse transcription kit (Promega, Madison, WI, USA). The mRNA expression was performed with the Quantifast SYBR green PCR kit (Takara) according to the manufacturer’s instruction. The expression of genes was normalized to *Gapdh*. The primers were: *Gapdh* Forward (5’-CTGGAGAAACCTGCCAAGTA-3’), Reverse (5’- AAGAGTGGGAGTTGCTGTTG-3’). *Opn* Forward (5’-AGAACCTCCAGGACGACTTTG-3’), Reverse (5’-TCACAATGCTTCTCCGCATCT-3’); *Acta2* Forward (5’-GTCCCAGACATCAGGGAGTAA-3’), Reverse (5’- TCGGATACTTCAGCGTCAGGA-3’); *Col1a1* Forward (5’-CATGTTCAGCTTTGTGGACCT-3’), Reverse (5’-GCAGCTGACTTCAGGGATGT-3’). qPCR measurement for variance were performed based on the ∆Ct values. The fold change is presented and normalized to the control group, setting the control comparative group to 1.

**4. Isolation and identification of adipose-derived mesenchymal stem cells (ASCs)**

ASCs were isolated from subcutaneous adipose tissue and differentiated as described previously. Briefly, ASCs were digested with 0.1% collagenase I (Gibco) for 1 hour at 37°C and filtered through a 70 mm nylonmesh. Isolated ASCs were cultured in low glutamine DMEM containing 10% FBS, 100 U/ml penicillin, and 1%PS. Nonadherent cells were removed following 24 h.

As for the adipocyte differentiation, ASCs were cultured in medium containing isobutylmethylxanthine, indomethacin, dexamethasone, and insulin. The presence of adipocytes was verified by staining for triglycerides with Oil Red O. For osteogenic differentiation, ASCs were cultured in medium supplemented with dexamethasone, L-ascorbicacid, and b-glycerophosphate. These cells were stained with Alizarin Red S to identify calcium deposition. All chemical reagents for differentiation were purchased from Sigma-Aldrich (St. Louis, MO, http://www.sigmaaldrich.com).

**5. Purification and electroporation of exosomes**

Exosomes were collected and electroporated with siRNA according to our established protocols (1). ASCs were obtained from subcutaneous adipose tissue and isolated according to our established protocol. Medium was changed every 3 days. Passage 4 to 6 ASCs were used for exosomes collections. Exosomes were purified by differential centrifugation processes according to our established protocols (2, 3). Briefly, Supernatant was collected and centrifugated at 800 x g for 5 minutes and then 2,000 x g for 10 minutes. Filtered supernatant was centrifuged at 28,000 x rpm for 3 hours at 4°C. The pellet was diluted in 100 µl PBS. Nanoparticle tracking analysis (NTA) was used for verify the concentration and size of exosomes. The siRNAs were purchased from RIBOBIO (https://www.ribobio.com/). For electroporation, the siRNA-exosomes mixture was electroporated at 400V, 125μF and ∞ ohms.

**6. EdU incorporation assay**

The effect of iExosomes on primary HSCs proliferation were determined by 5-Ethynyl-2'-deoxyuridine assay using BeyoClick^TM^ EdU-488 Kit (Beyotime, China). Briefly, after culturing with medium containing iExosomes for 24 hours, 10 μM EdU was added. After 2 hours' culture, HSCs were fixed with 4% paraformaldehyde, and then incubated with click additive solution for 30 min in the dark at room temperature. Next, cells were subjected to nuclear staining with DAPI for 5 min and finally observed by confocal microscopy.

**7. Haematoxylin and eosin staining and sirius red staining**

Liver tissues were fixed in 4% paraformaldehyde solution and embedded in paraffin. For each slide, the numbers of degenerated and necrotic hepatocytes were counted by the count tool of Adobe Photoshop 7.0. Hepatocytes were considered as necrotic according to condensation and dark staining of the cytoplasm and absence of nucleus (4). Hepatocytes degeneration was determined as previously reported by cell enlargement and swelling (5).

As for Sirius red staining, liver sections were stained according to manufacturer's protocol. We used counting grids in Adobe Photoshop 7.0 to analyze from each mouse. The percent area of fibrosis was measured as previously described (6).

**8. Serum alanine aminotransferase (ALT), aspartate aminotransferase (AST) and OPN measurement**

Mouse blood was collected from heart. The blood samples of healthy and liver cirrhosiss patients were collected from Tongji hospital in anticoagulant tubes. Serum was then immediately isolated by centrifugation at 4°C for 15 min at 3,000 rpm. Informed consent was obtained from all people. Measurements of ALT and AST were performed by the department of clinical laboratory in Tongji hospital. Measurements of OPN were performed by Shanghai Weiao Biotechnology.

1. **Hepatic hydroxyproline measurement**

The liver lysates were used to measure hydroxyproline contents using a hydroxyproline detection kit according to the manufacturer’s instructions (Jiancheng Institute of Biotechnology, Nanjing, China).

**Supplementary Figures**

**Supplementary Figure 1**


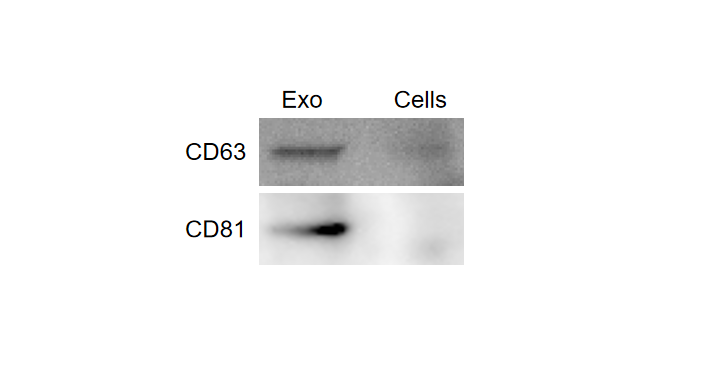


**Fig. S1** CD63 and CD81 were measured by western blot in exosomes and ASCs.

**Supplementary Figure 2**


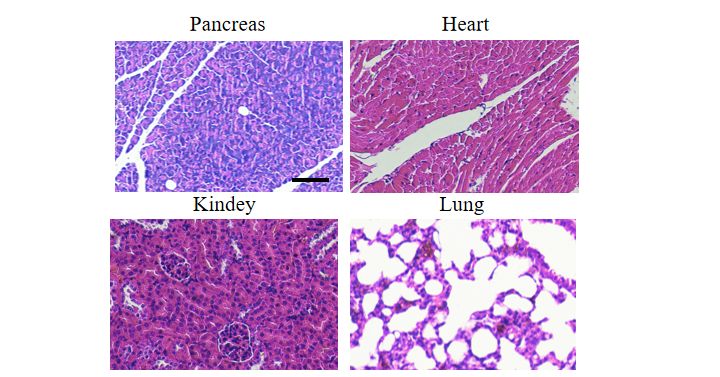


**Fig. S2 Exosome treatment did not cause observable cytotoxicity to other organs.** H&E staining of pancreas, heart, kindey and lung in mice treated with 1 billion exosomes.

**Reference:**

1. Tang M, Chen Y, Li B, et al. Therapeutic targeting of STAT3 with small interference RNAs and antisense oligonucleotides embedded exosomes in liver fibrosis. FASEB journal : official publication of the Federation of American Societies for Experimental Biology 2021;35:e21557.

2. Lorena D, Darby IA, Gadeau A-P, et al. Osteopontin expression in normal and fibrotic liver. altered liver healing in osteopontin-deficient mice. Journal of hepatology 2006;44:383-390.

3. Tang M, Jiang Y, Jia H, et al. Osteopontin acts as a negative regulator of autophagy accelerating lipid accumulation during the development of nonalcoholic fatty liver disease. Artificial cells, nanomedicine, and biotechnology 2020;48:159-168.

4. Krishna M. Patterns of necrosis in liver disease. Clinical liver disease 2017;10:53-56.

5. Lackner C, Gogg-Kamerer M, Zatloukal K, et al. Ballooned hepatocytes in steatohepatitis: the value of keratin immunohistochemistry for diagnosis. Journal of hepatology 2008;48:821-828.

6. Whittaker P, Kloner RA, Boughner DR, et al. Quantitative assessment of myocardial collagen with picrosirius red staining and circularly polarized light. Basic research in cardiology 1994;89:397-410.
